# Delivery of multiple ecosystem services in pasture by shelter created from the hybrid sterile bioenergy grass *Miscanthus* x *giganteus*

**DOI:** 10.1038/s41598-019-40696-2

**Published:** 2019-04-03

**Authors:** Christopher P. Littlejohn, Rainer W. Hofmann, Stephen D. Wratten

**Affiliations:** 1 Bio-Protection Research Centre, PO Box 85084, Lincoln University, Lincoln, 7647 New Zealand; 2 Faculty of Agriculture and Life Sciences, PO Box 85084, Lincoln University, Lincoln, 7647 New Zealand

## Abstract

The benefits of shelter in increasing crop yields and accelerating ripening has been well researched in fruit, arable and horticultural crops. Its benefits to pasture, despite its importance for livestock production, is less well researched. In this work, *Miscanthus* shelterbelts were established on an intensively irrigated dairy farm. Seven key ecosystem services associated with these belts were identified and quantified. Pasture yield and quality were recorded in *Miscanthus*-sheltered and control field boundaries with little shelter. Pasture yield increased by up to 14% in the sheltered areas downwind of *Miscanthus*. Pasture quality was equivalent in the sheltered and open areas. *Miscanthus* provided more favourable nesting sites for bumblebees and for New Zealand endemic lizards (skinks) compared to field boundaries. The sheltered areas also had higher mineralisation rates of organic matter and higher numbers of earthworms. Using a high-yielding sterile grass such as *Miscanthus* to deliver a wide range of ecosystem services also produced a bioenergy feedstock. In conclusion, full benefits of shelterbelts to the farming system cannot be fully assessed unless direct and indirect benefits are properly assessed, as in this work.

## Introduction

The expansion of intensive agricultural production, with increasing inputs per unit of land^1^, has been the prime means of meeting the needs of an ever-increasing world population^2^. This is likely to continue with predictions of an estimated increase of 70% in worldwide agricultural production needed in order to meet an estimated population size of 9.6 billion^3^ by 2050^4^. However, the consequences of this are, along with food waste and poor distribution, unsustainable systems of agricultural production, leading to low and degraded ecosystem services (ES)^5–7^. This state of agriculture is susceptible to perturbations, including those associated with climate change^8^.

Shelterbelts are linear plantings of shrubs or trees, used in the study area, on the Canterbury Plains of the South Island of New Zealand, to protect soil from wind erosion. However, they are important components of sustainable farming systems due to their ability to deliver a number of ES (Table 1), and are undervalued in intensive production systems. Consequently, their use has been declining^9^. The removal of shelterbelts, either through progressive deterioration, a lack of funding or incentives to replace them, or from active removal due to changes in farming practices, reduces the ability of the farming system to deliver ES^10^.

**Table 1:** Delivery of ecosystem services from a *Miscanthus* x *giganteus* (Mxg) shelterbelt on an irrigated, intensively run, dairy farm.

Classes of ecosystem services	Ecosystem functions	Ecosystem services that may result from the presence of Mxg shelter
Provisioning	Increased pasture biomass	Increases in feed supply for dairy cows
	Shelterbelt growth	Production of biomass used for bedding, feed, energy production
Regulatory	More bees	Increased pollination of local crops
	Reduced evapotranspiration	Improves the efficiency of water utilisation
	Increased mineralisation rate	Mineralisation releases nitrogen for crop use reducing the need to apply extra nitrogen
	Increase in earthworm biomass	More earthworms and soil organic matter and better drainage
Cultural	Improved habitat for vertebrates and beneficial invertebrates	Higher populations of endemic reptiles delivering conservation value

In this study an on-field measurement of ES delivered by re-instating shelterbelts on intensively irrigated dairy farms was performed. The shelterbelts were created from planting *Miscanthus* x *giganteus* Greef et Deu (Mxg; Poaceae), which has proven value as a feedstock for renewable liquid fuels and as a heat source^9^. The study area has seen in recent years an upsurge in the conversion of arable and dryland sheep and beef farms to dairy production. Until recently, this has largely been due to the increased profitability of dairy farming and the increase in availability and use of irrigation. The preferred method for the latter is centre-pivot irrigators with low ground clearance, requiring woody shelterbelts to be removed. The result is that extensive areas of the Canterbury Plains are now comprised of a flat, treeless landscape of low-diversity pasture. Consequently, the production system is low in ES provision, the most obvious example of which is low aesthetic value, which creates a public perception of unsustainable dairying^11,12^. The rate of generation of ecosystem dis-services, including water pollution, is also high^13^.

## Methods

### Study area

**Figure 1:**
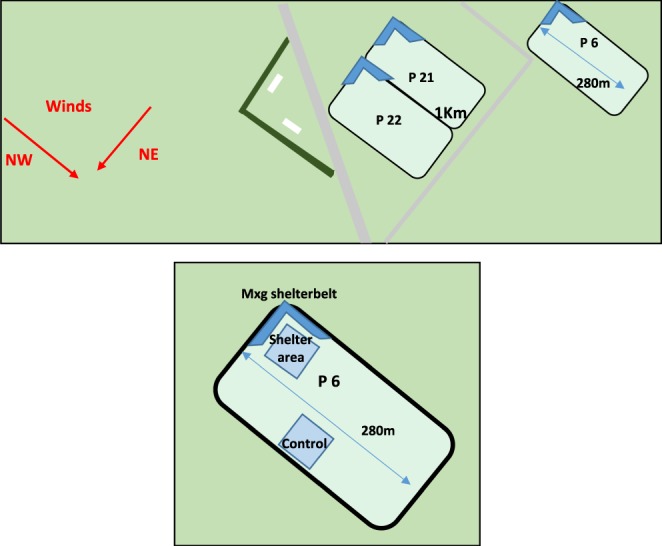
Location of three of the *Miscanthus* (Mxg) shelterbelts at Aylesbury farm and an enlarged view of field 6, showing the location of shelter and control areas.

The study was based at Aylesbury Dairy Farm (latitude −43.54, longitude 172.27; altitude 120 m), a supplier to Westland Milk Products Ltd in the province of Canterbury, New Zealand. The soil component of the stony study area comprised silt loams, such as Lismore and Chertsey loams. At the start of the study in 2012, the 150 ha, centre-pivot irrigated farm was in its first year of conversion from dryland sheep farm to intensive irrigated dairy farm. The cows were stocked at 5.7 ha^−1^ with 30% of the diet fed as a supplement consisting of silage (lucerne, maize and pasture) and vegetable waste. Three fields were planted in their northern corner with a 40 m by 40 m L-shaped Mxg shelterbelt which enclosed on two sides a 1600 m^2^ area. An equivalent sized control area in each field allowed measuring of shelter effects compared to no shelter at all times during the trial. The location of the control was based on the following: of sufficient distance from the Mxg not to experience shelter effects; under the same section of the centre pivot as the Mxg; of similar aspect; away from water troughs and field gateways; next to and incorporating the existing field boundary which comprised of a two-strand electric fence (Fig. 1). The primary aim of planting was to provide protection in the summer months from the drying northerly föhn winds that are a predominant feature of the Canterbury Plains. Plants were established as six single rows 1 m apart with a within-row spacing of 1 m, which gives a density of 10,000 plants ha^−1^. These areas were planted into newly sown ryegrass/white clover swards. The advantage of using Mxg as the shelterbelt plant was that, due to its rapid growth, it gained sufficient height by the second season to enable the ES delivery of the shelterbelt to be measured. Each winter, Mxg senesces and can be harvested, so the shelter effects of this type of shelterbelt are designed to be most prominent during late spring and summer, less so, due to senescence, in the winter. Non-harvested material can still provide some degree of shelter during the early spring while regrowth occurs. No pre-existing shelter was present on the farm prior to conversion.

Seven ecosystem functions were measured to assess their rate of change once shelterbelts had been re-introduced (Table 1) and five of these (bioenergy feedstock yield, increased pasture yield, improved soil mineralisation rate of organic matter, increased soil formation and improved irrigation efficiency) were given estimated financial value resulting from the extent of their improvement, so were ES. To estimate Mxg yield the heights of 10% of Mxg plants within each of the three shelterbelts planted were measured at the end of each growing season to estimate potential future yield. Maximum yield is not usually achieved until 4–5 years after planting but performance in the first season is very predictive of future yield potential^14^. There being a close relationship between height gain and DM yield for this plant species^14^.

### Changes in pasture production between sheltered and control areas

**Figure 2:**
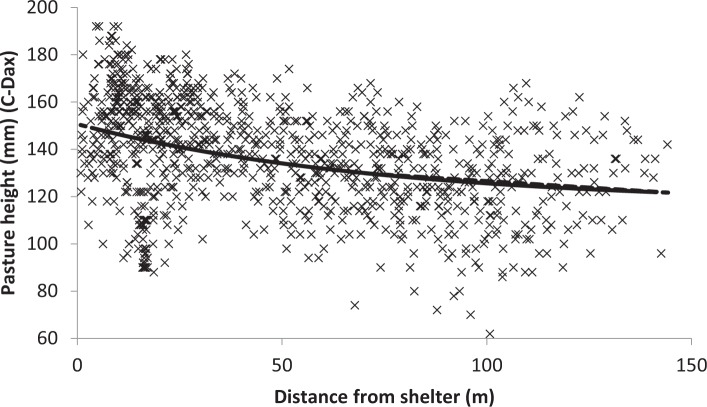
The effect of shelter on pasture height in Field 21 on 25 February 2014. Pasture height = 117 (±8) + 33.7 (±6.3) × 0.9865 (±0.0061) distance, where each value in brackets is the SE of the fitted coefficient.

Pasture height readings were taken with a C-Dax pasture meter (www.cdax.co.nz) which is pulled by a vehicle and continually records pasture height using a series of LED emitters and receivers mounted on two vertical bars. Mean readings/second were calculated to give data points with a GPS co-ordinate which can be mapped^15^. These measurements in control and shelter areas were achieved by towing a C-Dax in a spiral of continuously decreasing diameter around each 40 m by 40 m control and shelter area. Less intensive measurements from the rest of the field were also recorded by towing the C-Dax along the length of the field in rows 10 m apart so that a whole field yield map could be produced (Fig. 2). Over the 3-year study period, pasture height was measured the day before the cows grazed each field. Fields were rotationally grazed, cows spending 1 day in each field on average every 24 days during spring and summer. Mxg shelter height at the end of the growing season 1, 2 and 3 was 1 m, 2 m and 3 m, respectively. The shelter senesced over the winter and slowly degraded, since it was never harvested, until being replaced by new growth in the spring. Although pasture dry matter (DM) yield can be determined from pasture height readings using calibrated tables (www.cdax.co.nz), the basis of comparisons between field areas was made on a pasture height basis only. Differences in pasture plant species composition would alter the DM calculation when based only on pasture height. However, pasture plant species composition did not change over the 3-year study period.

Second-season shelter growth began in September 2013 and plants had reached 1 m in December 2013, 2 m in February 2014 and 2.8 m in April 2014. This rapid growth rate should have enabled shelter effect on pasture production to be analysed throughout the second season. However, a storm in September 2013 collapsed the centre-pivot and consequently pasture growth was influenced more by lack of irrigation than shelter. Irrigation did not re-commence until December 2013 and any shelter effect was not evident until February/March 2014. Shelter effect in season two was thus investigated by whether a shelter-distance effect on pasture height existed as replicate numbers had been reduced and variation in soil available water capacity (AWC) had been compromised by the lack of irrigation and is affected by soil textures. In season three, shelter comparison was made by comparing yield differences between shelter and control areas in each field.

### Measuring evapotranspiration rates in control and sheltered areas

Variation in stomatal conductance of red and white clover and modelling differences in evapotranspiration (ET) rates^16^ was carried out in this work. Clover was used as it constitutes 30% of the pasture plant mix and its wider leaf enables more accurate measuring to be performed. Single-leaf stomatal conductance was measured on cloud-free days using a hand held SC-1 Decagon leaf porometer (www.decagon.com). Measuring took place in early summer 2014, and 2015 in the control and Mxg sheltered areas. Between 10.00 h and 14.00 h the stomatal conductance of the second unfolded leaf of each of five white clover plants was measured in control and sheltered areas. Plants selected were within an area 5 to 20 m out from the Mxg or field edge, respectively, and 10 m in from the Mxg shelter ends. Measuring alternated continuously between shelter and control areas in the same field over the 4-hour period.

Mobile weather stations were designed and built to enable the necessary data to be collected to calculate ET using the in-built Campbell data logger calibration. This uses an estimated ET rate based on the American Society of Civil Engineers (ASCE) standardised Reference Evapotranspiration equation^16^. ET rates were measured in control and shelter areas of each field for one 2-week period immediately pre-grazing in the early summer of the third year of the trial when shelter was above 2.5 m. At the same time, soil moisture levels were recorded using two methods. Soil water potential was measured using one heat dissipation matric potential sensor (Campbell Scientific Inc., model 229_L) placed at a depth of 20 cm. Soil volumetric water content was measured using two water content reflectometers (Campbell Scientific Inc., model CS616) inserted into the soil. All soil moisture sensors in one field were positioned within a 1 m^2^ area at a distance 10 m from the middle of the Mxg shelter.

### The influence of Mxg shelterbelts on the mineralisation rate of soil organic matter

Mineralisation rate in areas sheltered by Mxg plants at the end of their second and third season was measured in fields 6, 21 and 22. Mean shelterbelt height at the time of sampling was 1.8 m and 2.4 m.

Measurement of mineralisation rate involved using strips of rigid plastic 16 cm long, 0.6 cm wide and 1 mm thick with 16, 2-mm holes drilled along the strip^17,18^. These were filled with a paste that comprised water and (by weight): cellulose (65%), agar (15%), bentonite (10%) and wheat bran (10%). This mixture simulates the key constituents of dead plant material on or in the soil^19^. When the paste had dried, four test probes were placed at each location 6, 12, 26 and 40 m out from the shelter or fence line in each shelter and control area.

Soil microorganisms and invertebrates consume the ‘bait’ and the number of holes that are empty (partially or fully) gives a relative measurement of the rate of mineralisation^20,21^. Control probes were placed in all areas near to the test probes. These were removed after 10 days and a further one removed every 3 days until signs of bait removal were detected. Once bait removal had been detected in any of the control probes two of the four test probes for recording bait removal rate were removed from all sampling points. The remaining two probes were removed 7 days later. A mean was calculated for the number of holes with evidence of being consumed over the four probes and used to calculate proportional mineralisation rate. This showed differences in rates between sheltered and control areas and with distance out from the shelter or field boundary. These values were also used to estimate the economic value of the mineralised N provided. Total organic matter content in the fields was calculated using the bulk weight of soil and total N values obtained from soil testing results and using the assumptions, as used in Sandhu *et al*.^22^, that the ratio of organic matter to N is 20:1. The economic value of plant nutrient mineralisation provided by soil microorganisms and invertebrates was assessed using the method outlined in Sandhu *et al*.^22^. The total amount of N mineralised was estimated from Eq. (1) and valued at the equivalent price of 1 kg N = US$0.49 kg^−1^ for 2015 (http://www.ravensdown.co.nz).

1 $${{\rm{N}}}_{{\rm{\min }}}={\rm{n}}\times {\rm{b}}\times {\rm{v}}\times {\rm{k}}\,{10}^{-3}$$

where N_min_ = amount of nitrogen mineralised, n = total amount of nitrogen (%) in soil, b = bulk density of soil (g cm^−3^), v = volume of soil (cm^3^), and k = percentage mineralisation (%).

Soil quality results collected at the start of the study and the differing mineralisation rates between shelter and control areas over six fields in 2014 and 2015 were used to estimate the economic value of mineralisation of organic matter N content.

### The influence of Mxg shelterbelts on earthworm populations

Earthworm populations were assessed to compare Mxg-sheltered and unsheltered field areas. Two samples were taken in April 2014 and April 2015, respectively. Three spade digs were taken at each sampling location (at 0, 6, 12, 26 and 40 m) as used for measuring mineralisation rate. Samples were sorted *in situ*, earthworm numbers recorded and a mean value for each sampling point was calculated.

The economic value of earthworms in soil formation in this work was calculated based on the assumptions that the mean earthworm biomass is 0.2 g per worm^23,24^ and 1000 kg of earthworms forms 1000 kg of soil ha^−1^ yr^−1^ ^25^ as presented in Sandhu *et al*.^22^. The price of topsoil in New Zealand is around US$33.75 per tonne (http://www.aucklandlandscape.co.nz). From these assumptions and the above economic information, the annual value of soil formation by earthworms was calculated.

### Nesting densities of bumblebees in Mxg and field edges

In November 2012, four artificial domiciles, as used by Barron *et al*.^26^, were placed along the unsheltered field edge and four within newly planted Mxg shelterbelts in three fields. These domiciles each had four nesting compartments with entrance holes alternating on each side of the box (Barron *et al*.^26^). In the present study, bumblebee occupancy of nesting chambers was recorded if any attempt at nest founding was made. This follows the definition of Donovan & Weir (1978) and Pomeroy (1981) used in Barron *et al*.^26^. Domiciles were monitored during spring and summer for the 3 years of the study.

### Lizard (skink) occupancy of refuges in Mxg and field edges

In January and February 2015, 96 pieces of corrugated roofing material (Onduline; www.onduline.com) were used as potential skink refuges to determine skink presence. For each refuge, two sheets of 40 cm × 40 cm Onduline^27^ were stacked, with small spacers (10 mm diameter wooden dowels) between them. Sixteen of these refuges were placed in Mxg shelterbelts in their third season and 16 in a line along an unsheltered field edge of each of three fields. Refuges were placed 3 m apart as recommended by Lettink^27^ as a suitable spacing for monitoring populations of unknown size.

Refuges were in place for a settling period of 5 months before monitoring began. Sampling was carried out on 22 and 30 January and 5 and 17 February 2015. They were inspected between 06.00 h and 07.00 h when skinks were still cold and immobile. Upper and lower Onduline layers were raised to check for skink presence within and below each refugium and then returned to their original position. For the artificial retreats, total skink encounter numbers were added up over four monitoring sessions, and a 95% confidence interval for true mean effect of shelter on skink numbers per refuge calculated to see if there was a significant difference in encounters from different habitats.

### Statistical analysis

**Figure 3:**
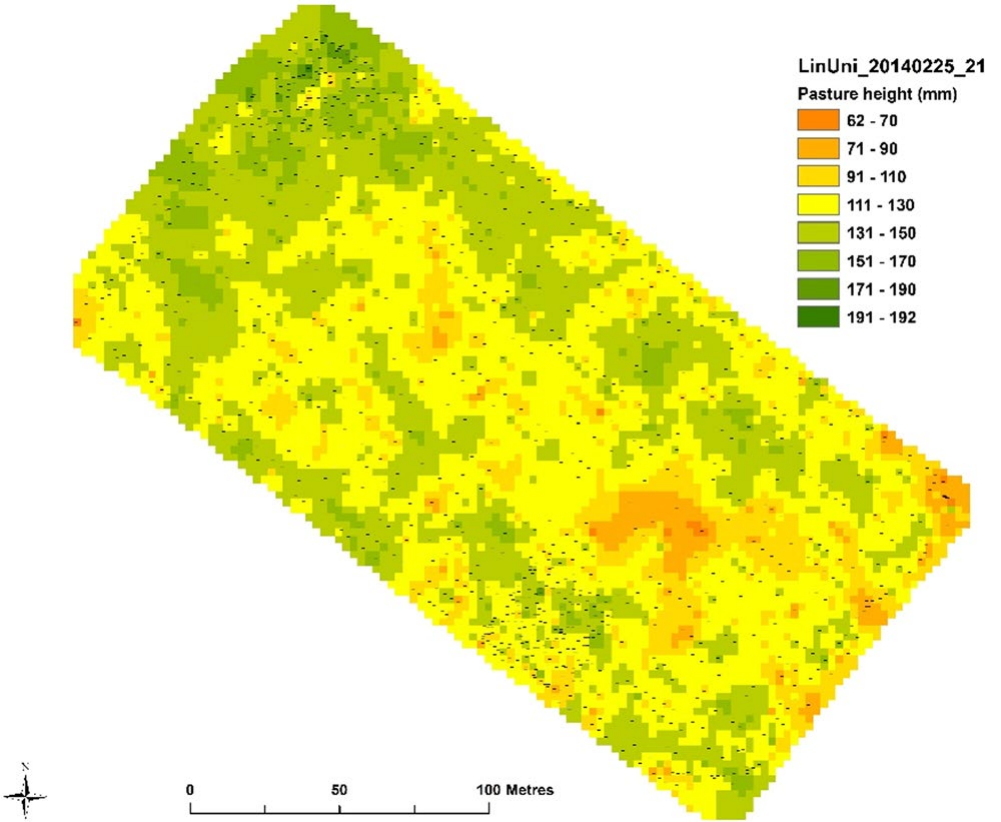
Yield map showing increased pasture height, in field 21 on 25 April 2014, in the field area sheltered from northerly winds by *Miscanthus*.

Pasture yield difference between shelter and control areas were determined by analysis of yield differences indicted by yield maps produced using Arcmap 10.1. For 2013 to 2014 season an exponential regression analysis using GenStat 16 (Fig. 3) was conducted. This nonlinear regression was conducted for six pre-grazing pasture height measurements, three from each of two fields, where a shelter response, as indicted by yield maps produced with ArcMap 10.1 (Fig. 2), was evident. For the 2014 to 2015 season an Anova using Genstat 16 was conducted on all collected C-dax readings to determine if shelter was affecting pasture yield.

Anova using Genstat 16 was also used to show whether differences in stomatal conductance between shelter and control areas was significant.

A chi square test of skink refuge and bumblebee domicile occupancy was performed to assess if there was any significant difference between occupation rate in shelter and control areas.

## Results

### Biomass yield from Mxg shelterbelts

At the end of the first season mean plant height was 98 cm (σ = 15) for shelter one, 102 cm (σ = 14) for shelter 2 and 79 cm for shelter 3 (σ = 12) and plant survival rate was over 90% in all shelterbelts placed under the centre pivot. Expected height for a healthy high yielding crop would be 100 cm at the end of growing season one. Stand height is reflective of yield and expected height to achieve over 30 tDM/ha would be 3 m by April 2015^14^. All shelterbelts had reached 2.5 m by February 2015. Therefore, it is highly likely that yields of at least 30 t ha^−1^ yr^−1^ are attainable under irrigation on the Canterbury Plains. Height gain of Mxg shelterbelts under irrigation over the three seasons was consistent with that required for high DM yields.

### Pasture yield response to shelter

The shelter distance analysis showed that for all six pre-grazing measurements analysed, pasture height decreased with distance from the shelter (P < 0.001). For control areas, there was no significant effect of distance for any of the six pre-grazing periods analysed. For these measurements the mean C-Dax pasture height for the shelter area was significantly (P = 0.05) higher in sheltered areas (138 mm shelter, 120 mm control). Using the standard C-Dax calibration for Canterbury, New Zealand, this equates to 3245 kg DM ha^−1^ for pasture influenced by the shelter and 2901 kg DM ha^−1^ for control areas, an increase of 12%.

**Figure 4:**
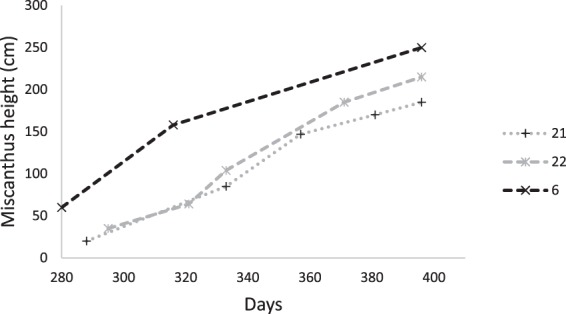
Increases in height of new growth from three *Miscanthus* shelterbelts in their third season, 2014–2015, in fields 6, 21 and 22 at Aylesbury farm (day 365 = 31 December 2014).

In season three, increases in pasture height in Mxg sheltered areas compared to control were detected across all three fields studied. This was not significantly dependent on Mxg shelterbelt height but was significantly influenced (P = 0.04) by the month when data were collected. In November 2014 and January 2015, respectively, mean percentage increase in pasture height in sheltered areas was 11.7% and 16.2%, compared to the control. In October and December 2014, it was 5% and −9%, respectively. Mean shelter height in November 2014 was 0.85 m and in Jan 2015 was 2.01 m (Fig. 4). For the months where the pasture height was higher in the sheltered area compared to the control, this difference was significant (P = 0.05). Analysis of weather data showed that estimated ET rate of the pasture was significantly higher (P < 0.007) in months where yield increases were detected.

### The effect of shelter on the stomatal conductance rates of clover plants

Hand-held porometer readings taken between 10.00 and 14.00 h between January and February 2014 showed significant differences between stomatal conductance when the clover plants in the pasture were sheltered from drying northerly winds (Table 2). The differences in conductance were significant only when northerly winds were predominant and the mean increase of conductance over those dates was 35% which is a significant increase (P = 0.001).

**Table 2:** Differences in mean stomatal conductance between sheltered and control areas of fields 6, 21 and 22, during January and February 2014.

Field	Date	Mean stomatal conductance		One-way ANOVA
		mmol/(m² · s)		
		Shelter area	Control area	
6	07.01.2014	636	426	<0.001
6	14.01.2014	655	590	0.61
6, 21	17.01.2014	700	550	0.012
6, 21	29.01.2014	874	681	<0.001
6, 21	04.02.2014	1015	1000	0.73
21, 22	27.02.2014	784	622	<0.001

### The effect of shelter on modelled evapotranspiration (ET) rates and on soil moisture content

Calculations of the estimated ET rate (Table 3) using data collected by mobile weather stations and the Campbell ASCE standardised Reference Evapotranspiration Equation [16] during December 2014 and January 2015, showed that ET rates were reduced significantly (P = 0.05) in sheltered areas. Mean ET rate being 20% lower in sheltered areas. This was at a time when irrigation rate was not keeping up with ET rate, when soils were beginning to dry and hot northerly winds were frequent.

**Table 3:** Evapotranspiration (ET) rates in shelter and control areas in fields 6, 21 and 22 in December 2014 and January 2015.

Field	Area	Campbell total ET (mm)	ET rate (mm) day^−1^	% reduction in ET	Dates
6	Shelter	63.0	7.0	23.2	22.12.14–31.12.14
	Control	82.0	9.1		
21	Shelter	94.0	6.0	20.0	31.12.14–15.01.15
	Control	116.0	7.5		
22	Shelter	120.0	10.9	16.2	20.01.15–31.01.15
	Control	142.0	13.0		

Soil plant-available water content was monitored using two volumetric water content (Ɵv) probes and one soil water tension (SWT) probe. Results from fields 6, 21 and 22, collected at the same time as the ET measurements, showed that Ɵv in all fields was higher in the sheltered areas. In fields 21 and 22 this difference also correlated with lower soil water tension values. In field 6, differences in soil water tension were not detected as Ɵv in the control area, despite being lower than in the shelter area, was still above 27%. On-farm soil testing results showed this was at a level where plants will not suffer water stress. The sheltered areas had higher soil moisture concentrations than control areas. Since both areas were under the same section of the pivot irrigator, this indicates that ET rates were higher in the control, as irrigation rates in each area were the same. Local variations in soil texture could be involved in variations in soil water availability, but overall, sheltered areas did have higher negative levels.

### The effect of shelter on mineralisation rate of soil organic matter

The total amount of N mineralised was estimated and valued at the current estimates of N/kg soil (Table 4). The results show an increase in value of 35% in 2014 and 60% in 2015 in sheltered compared to control areas.

**Table 4:** Mean rate of mineralisation of soil organic matter (OM) and calculation of its potential economic value in sheltered and control areas in fields in 2014 and 2015 (methods based on Sandhu *et al*.^20^).

Sampling year	Shelter area	Rate of mineralisation (%)	Mineralised OM ha^−1^ (×10^3^ kg)	Mineralised N available ha^−1^ yr^−1^ (kg)	USD value of mineralised N @ US$0.49 kg N (US$ ha^−1^ yr^−1^)
2014	Shelter	40	27.94	1397	1676.4
2014	Control	30	20.6	1030	1236
2015	Shelter	64.8	45.26	2263	2715.8
2015	Control	40.6	28.36	1418	1701.5

For all shelter areas in each sampling year: bulk density = 1.27 g/cm^3^; weight of soil ha^−1^ 10 cm deep^−1^ = 12.7 × 10^3^ kg; total OM = 5.5%; total OM ha^−1^ = 0.6985 × 10^3^ kg.

### The effect of shelter on earthworm abundance and value in pasture

Table 5 shows the estimated economic value from soil formation from earthworm activity in sheltered areas was 57% higher in 2014 and 46% higher in 2015. The mean value of all areas was $35.4 ha^−1^ yr^−1^ in 2014 and only $6.54 ha^−1^ yr^−1^ in 2015. The difference due to the much drier soil conditions at the time of sampling in 2015.

**Table 5:** Earthworm populations, biomass and economic value of soil formation in *Miscanthus* x *giganteus* sheltered and control areas (2016 values).

Sampling year	Shelter area	Earthworm number/m^2^	Earthworm biomass (t ha^−1^)	Soil formation (t ha^−1^ yr^−1^)	Soil formation value (US$ ha^−1^ yr^−1^)
2014	Shelter	728	1.456	1.456	49.36
2014	Control	315	0.63	0.63	21.36
2015	Shelter	125	0.25	0.25	8.47
2015	Control	68	0.136	0.136	4.61

### Domicile colonisation rates by bumblebees in Mxg shelterbelts and unsheltered field edges

Of the four species of introduced bumblebees in New Zealand, only *Bombus hortorum* and *B*. *terrestris* occupied the domiciles in the study area. Only the refuges placed within the Mxg shelterbelts were occupied. Occupancy rate between the Mxg shelterbelt and those placed below the unsheltered fence line showed a significantly higher colonisation rate in the Mxg shelter (P < 0.01). In year 3 of the trial, 29% of the domiciles placed in the Mxg were occupied.

### Assessment of lizard shelter in Mxg shelterbelts and field edges

Skink species were recorded occupying only the refuges in Mxg shelterbelts and none was found in refuges placed along electric fence-lines marking field boundaries. The skink species was either the New Zealand common skink (*Oligosoma polychroma* Patterson & Daugherty) or McCann’s skink (*O*. *maccanni* Hardy), the separation of these two species is difficult^28^.

## Discussion

### Bioenergy feedstock production

With yields of at least 30 t ha^−1^ yr^−1^ attainable under irrigation, the Mxg shelterbelt has direct financial value as a bioenergy feedstock, without considering other ES delivery benefits from re-instating this form of shelter. Although the full benefits of producing a bioenergy feedstock on New Zealand farms will not be realised until the bioenergy industry further develops, the real energy value of producing this feedstock can be calculated. At present there are 220,000 ha of dairying in Canterbury, New Zealand. If 8% of this area is converted to Mxg shelterbelts, such as in the form of field-margin strips, then the production of Mxg at 30 t DM ha^−1^ yr^−1^ using 90 kg N ha^−1^ yr^−1^ would be 528,000 t DM yr^−1^ for Canterbury. This equates to 9,504,000 GJ of energy/year, based on the Mxg energy content being 18 MJ kg DM^−1^ ^14^. This GJ total has a value of US$115,714,194 at US$12.18 GJ^−1^ (2016 values; http://ramblingsdc.net/EnCalcs.html). A loss of 8% of land used to produce pasture if yielding 18.5 t DM ha^−1^ yr^−1^ from 200 kg N ha^−1^ yr^−1^ ^29^ would equate to a reduction of 325,600 t of pasture DM or 23,257,143 kg milk solids (MS) yr^−1^ at a ratio of 14:1 DM consumed to MS produced (http://www.sciquest.org.nz/node/40615). At US$3.6 kg^−1^ MS, the present low value of MS gives US$83,260,571 (2016 values), representing a net gain of US$32,453,622 in the Mxg plots. If the energy content of Mxg can be harnessed efficiently, its use as a shelterbelt plant can negate, during times of low world milk price, the cost of the pasture production lost from using land for shelter. This excludes net gains from increased pasture yield and true-cost accounting, including internal and external farm ES and the possibility of enhancing Mxg yield considerably by inoculating the plants with the beneficial fungus *Trichoderma* spp^30^.

### Reduced evapotranspiration rates

Measuring parameters linked to pasture water stress showed that plants in field areas protected by Mxg shelterbelts from drying northerly winds exhibited decreased signs of water stress. This was apparent from manual porometer readings of the stomatal conductance of clover plants and calculations of the estimated ET rate using the ASCE standardised Reference Evapotranspiration equation. All sheltered areas also had higher volumetric soil moisture levels (Ɵv) than control areas. Both were under the same section of the centre pivot and there was no rainfall during the measuring period, so this supports ET rates being higher in the control areas. The implications of this are wide ranging when considering the high monetary and environmental costs of irrigation and the need to maximise water use efficiency to maximise economic yield. Mxg shelterbelts 4 m high can be successfully created under centre pivots without impeding pivot movement. Shelter distance is correlated to shelter height^31^ and the resultant sheltering effect would be expected to extend outwards to a distance of 10–12 times the shelter’s height^31^.

### Improved pasture yield

In the study area, a predominant feature of the climate is the dry summers with drying northerly winds^32^. The shelterbelts created were designed to protect field areas from the effects of these and it is under these weather conditions that increases in pasture yield were shown. The results from the study showed that mean increases in pasture DM yield of 12% were attributable to shelter effect during periods of high evapotranspiration rates when northerly winds were frequent. The benefits shown here are likely to be more consistent if shelterbelts are planted around the whole field boundary. Thus a loss of 8% of land used to produce pasture in order to plant Mxg shelter does not result in an 8% reduction in pasture DM production as the pasture production lost is counterbalanced by a 12% increase in pasture growth from shelter effect. Pasture yield increases resulting from shelter are weather dependent and difficult to predict. However, they are highest when conditions are unfavourable, such as seen in Canterbury, New Zealand, during dry periods and persistent drying föhn winds.

### Improvements in soil function

The value of improved mineralisation rate of organic matter and soil formation was calculated according to methods outlined in Sandhu *et al*.^22^. The estimated mean annual value of available N from increased mineralisation rate resulting from shelter effect was US$1000 ha^−1^ in sheltered field areas next to shelterbelts in their third season (2016 values). The estimated mean value of improvements in soil formation from increased earthworm activity was US$28 ha^−1^ from shelterbelts in their second season. What is significant is that despite the recent creation of the shelterbelts and the small area they occupied in relation to the field in which they were placed, statistically significant increases in value of mineralisation were detectable. The value of soil formation was double in Mxg sheltered areas for both measuring periods. This is most likely due to the control areas being dryer as a result of higher ET rates. The single most important climatic factor affecting earthworm survival is moisture stress^33^. The activity of soil fauna in general is increased by elevated moisture levels provided soil is not cold and saturated^34^.

### Provision of habitat for bumblebees and skinks

Occupancy rates of bumblebee nesting motels and skink refuges were higher for those placed in the Mxg shelterbelt compared to those along the unsheltered fence line. Occupancy rate of motels of this type is generally low^35^, so the fact that any improvement was detected is an excellent outcome. The benefits of providing improved habitat for bumblebees, which are important pollinators, goes well beyond the internal functioning of the farming system^36^. The increase in the use of rest areas by skinks within the Mxg shelterbelt illustrates that ES cannot always be assessed purely on the basis of financial outcomes^37^. In relation to enhancing biodiversity, existence value and human wellbeing^37–39^ are valuable ES functions of the shelterbelt.

## Conclusion

The decision to reinstate or maintain existing shelterbelts in intensive agricultural systems is often perceived as a trade-off between land lost for production and financial and other benefits from having shelterbelts on the farm^9^. However, this study shows increases in pasture production resulting from shelter effect can be higher than that lost from reduced pasture production area. In addition, this study also shows that defined financial benefits can be attributed to a wide range of ES delivery from bioenergy shelterbelts, further enhancing its economic value. In the presence of a well-developed bioenergy industry, these bioenergy shelterbelts not only improve sustainability of the production system but also provide outputs comparable, if not greater, than those from shelter-less intensive systems. As well as these defined financial benefits, improvement in non-market and intrinsic values was also reported. Price does not equal value and an economics-only approach under-represents non-market and intrinsic values^40^. This study, together with work such as that of Sandhu *et al*.^41,42^ further strengthens the case for more diversified, ES-rich, integrated agricultural systems that enhance functional agricultural biodiversity, minimise expensive inputs and external costs and are less energy intensive.

## Data Availability

The datasets generated during and/or analysed during the current study are available from the corresponding author on reasonable request.
